# Risk factors for small for gestational age as defined by a birthweight z-score below minus one: A prospective observational study

**DOI:** 10.18103/mra.v12i8.5731

**Published:** 2024-08-29

**Authors:** Hein Odendaal, Lucy T Brink, Anusha Lachman, Daan Nel

**Affiliations:** a Department of Obstetrics and Gynecology, Stellenbosch University, Cape Town, South Africa; b Department of Psychiatry, Stellenbosch University, Cape Town, South Africa; c Department of Statistics and Actuarial Science, Stellenbosch University, Stellenbosch, South Africa

## Abstract

**Objective:**

To determine the maternal risk factors for small-for-gestational-age newborns as defined by a birthweight *z*-score (BWZS) < −1.0

**Design:**

A prospective cohort study with recruitment from August 2007 to January 2015.

**Setting:**

Recruitment at a community health centre with assessments at Tygerberg Academic Hospital, Cape Town, South Africa.

**Population:**

A largely homogeneous population in a low socioeconomic residential area in Cape Town.

**Methods:**

This study is a further analysis of the data of the Safe Passage Study which investigated whether exposure to alcohol and tobacco was associated with increased risk of stillbirth and sudden infant death syndrome (SIDS).

**Main outcome measures:**

Birthweight *z*-score < −1.0

**Results:**

Individual odds ratios (ORs), in descending order, were associated with smoking, drinking, and preeclampsia (2.45), previous stillbirth (1.85), smoking (including smokers only and drinkers who also smoked) (1.55), preeclampsia (1.52), smoking and drinking (does not include smokers only or drinkers only)(1.43), hypertension (1.28), drug use (1.24), drinking during pregnancy (including drinkers only and drinkers who also smoked) (1.18), thoughts of self-harm (1.13), and crowding (1.10). After multiple logistic regression, highly significant ORs were found for previous stillbirth (1.89), cigarette smoking (1.84), hypertension (1.40), education (0.94) and body mass index (BMI) (0.95). Thoughts of self-harm then had an OR of 1.08 (95% confidence interval (CI) 1.00–1.18).

**Conclusion:**

Previous stillbirth, cigarette smoking, hypertension, lesser education, and a lower BMI were associated with the highest risks for low BWZS.

## Introduction

About 20% of newborns in low- and middle-income countries (LMICs) are small for gestational age (SGA).^1^ As the detection rate of being SGA during pregnancy is poor,^2^ increased efforts are needed to identify all possible maternal risk factors to be used as a screening tool for SGA.

In other studies, of the same community, maternal vascular malperfusion was the second most common cause of stillbirth (17%), after placental abruption (26%),^3^ and birthweight was <10^th^ percentile in 25.6% of infant deaths in contrast to 17.7% of survivors.^4^

Maternal undernutrition contributes to poor fetal growth, more so in South Asia and sub-Saharan Africa than anywhere else.^5^ It has been suggested that childhood undernutrition starts during pregnancy, with implications that early intervention, during pregnancy, is needed to reduce SGA births.^6^

Traditional screening for high-risk pregnancies in LMICs focuses more on risks for maternal morbidity and mortality than on risks for SGA newborns. Socioeconomic conditions such as education and income are rarely referred to. Maternal depression or thoughts of self-harm are not included among risk factors,^7–10^ despite the evidence that many outcomes that contribute to maternal and child morbidity in LMICs have direct associations with maternal mental health, which is responsive to targeted intervention.^11^

As the use of late-pregnancy ultrasound scans to determine gestational age in LMICs still needs validation,^12^ it is unlikely that a fairly accurate diagnosis of SGA or fetal growth restriction (FGR) would be made in these countries. Therefore, other measures to best identify risks for SGA newborns in the community should be explored.

As various risk factors for SGA births play a role, a multifaceted approach to address SGA is advised, concentrating on population-attributable risks, since little is known about these.^13^ According to the latter study, the top three risk factor categories in sub-Saharan Africa are nutrition (25%), environment and other exposures (13%), and general health issues (11%). For South Asia the categories are nutrition (31%), environment and exposure (15%), and pregnancy history (12%).

As environmental and exposure conditions differ much between communities, for example the high rate of alcohol and tobacco use in the community where the index study was done,^14^ there is a need to determine the most appropriate risk factors for SGA for this community.

Valuable data collected during the Safe Passage Study (SPS)^15^ were therefore used for the present study to identify risk factors for SGA as determined by birthweight *z*-score (BWZS) < −1.0. Different maternal, psychosocial and environmental conditions and their combinations were investigated to identify newborns with a low BWZS (< −1.0).

## Methods

The SPS was a large prospective multidisciplinary study in South Africa and North and South Dakota to investigate the association of smoking and drinking during pregnancy with SIDS and stillbirth.^3,16^ For this index study, only the South African information of the SPS, collected between August 2007 and October 2016, was used to examine the association between potential maternal risk factors for SGA as assessed by low BWZS.^13^

Women were recruited for the SPS from those waiting for their first antenatal visit. As there were more women each day waiting to be seen than could be accommodated in the study, only a limited number of women were recruited at each session. There was no selection of any specific women.

Collected data included maternal age, parity, gravidity, thoughts of self-harm and postnatal depression score, education, crowding index, household income, maternal height, length, head circumference, mid-upper-arm circumference (MUAC), triceps skinfold thickness, body mass index (BMI), history of a previous stillbirth and self-reported use of cigarettes, alcohol or drugs.

Maternal history, demographic and anthropometric information were obtained at enrolment for antenatal care or at the first of three antenatal visits by well-trained and experienced research midwives. Women were weighed on calibrated high-quality scales. For the MUAC, the midpoint of the upper arm was first determined and then the arm circumference at this point. All measurements were done twice, each time starting right at the beginning of the procedure. If the two measurements differed by > 1 kg or 2 mm respectively, a third measurement was taken and the mean of the closest two measurements was used. Further details on the methods of the SPS are given in more specific publications.^4,17–19^ A score of more than 13 on the Edinburgh Postnatal Depression Scale (EPDS) was used for the diagnosis of depression, as described previously.^20^ After delivery a medical chart abstraction was done which included information on medical conditions and hypertensive diseases during pregnancy, gestational age at birth (as determined by an early ultrasound examination), birthweight and sex of the newborn.

Reference values of the INTERGROWTH-21^st^ project were used to assess SGA as they also considered the effects of gestational age and sex of the newborn.^21^ Mothers of newborns with a BWZS < −1.0 were compared with those who had newborns with a BWZS of −1.0 to 1.0. Maternal information when the BWZS was > 1.0 was not analysed. Low birthweight was defined as below 2500 g.

### Statistical analyses

Statistical analysis was done with STATISTICA (Dell Inc. Dell Statistica (data analysis software system), version 13. software.dell.com). Continuous variables were compared with nominal variables using analysis of variance (ANOVA). Relationships between two continuous variables were analysed by regression analysis. Maximum likelihood chi-square statistics were used to analyse associations between nominal variables. For dichotomous variables odds ratios (ORs) with confidence intervals (CIs) were computed. The relationship of continuous and dichotomous input variable(s) on a dichotomous output variable were analysed with logistic regression or multiple logistic regression.

## Results

A total of 5 207 newborns were assessed for BWZSs, of whom 1 558 (29.9%) had low BWZS. A profile of the participants is given in Table 1. The mean age of women was 24.4 years, the mean gravidity 2.1 and the mean duration of formal education 10 years. The prevalence rate of preterm birth was 13.3%, 20.2% of newborns weighed less than the 10^th^ centile for gestational age and gender, and.16.3% of newborns had a low birthweight.

**Figure 1:**
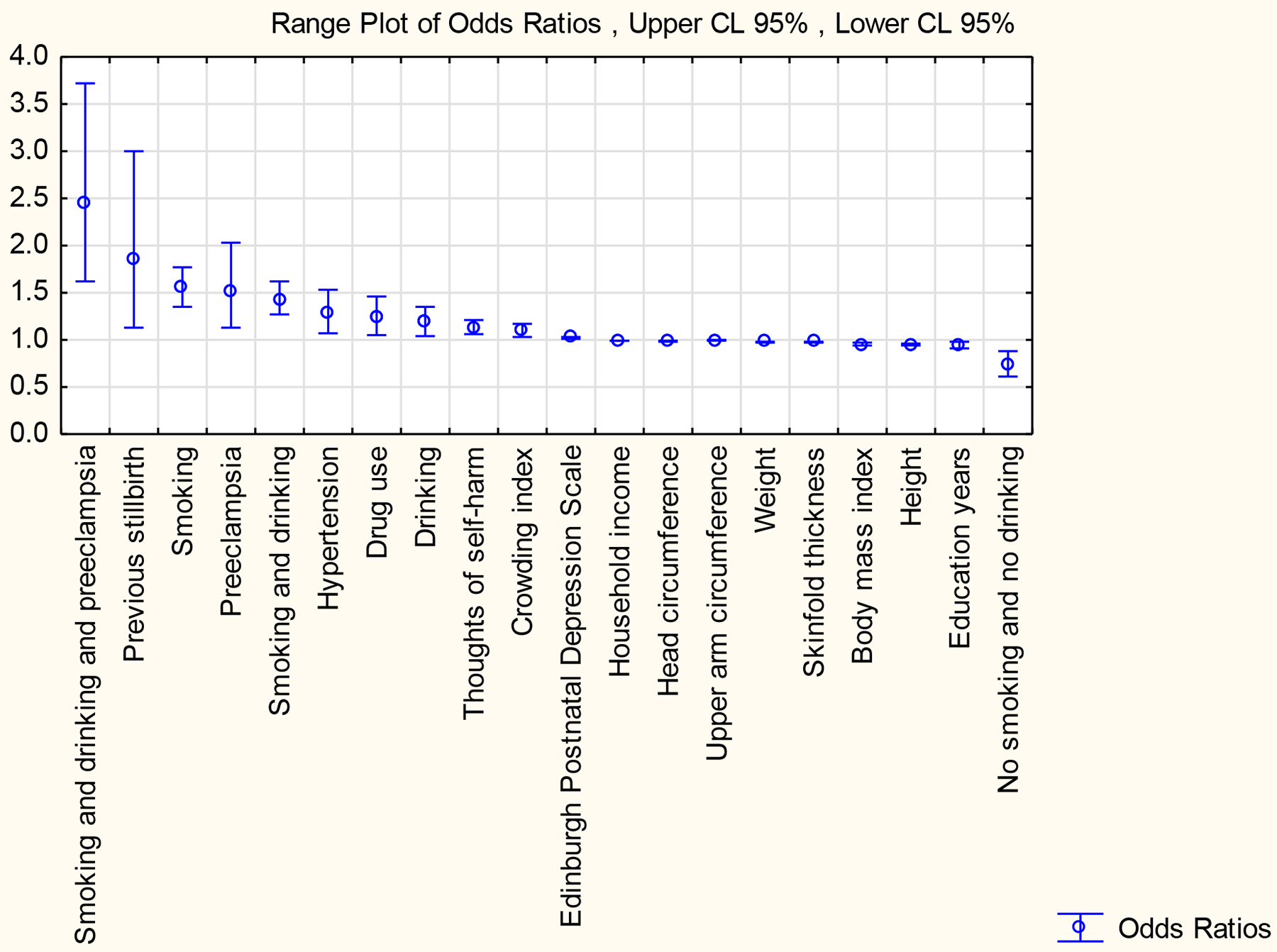
Range plot of significant odds ratios of maternal variables associated with low infant birthweight z-scores <−1.0

The ORs of all the associations with their CIs and significance with a BWZS < −1.0 are given in Table 2A, with only the significant associations portrayed in Figure 1. Increased ORs were associated with smoking, drinking and preeclampsia (OR 2.45), previous stillbirth (OR 1.85), smoking (includes smokers only and drinkers who also smoke) (OR 1.55), preeclampsia (OR 1.52), smoking and drinking (does not include smokers only or drinkers only) (OR 1.43), hypertension (OR 1.28), drug use (OR 1.24), drinking during pregnancy (includes drinkers only and drinkers who also smoke) (OR1.18), thoughts of self-harm (OR 1.13), crowding (OR 1.10) and a high EPDS (OR 1.02). Reduced ORs were associated with increased household income (OR 0.99), increased maternal head circumference (OR 0.99), increased MUAC (OR 0.99), increased maternal weight (OR 0.98), increased skinfold thickness (OR 0.98), increased BMI (OR 0.95), increased maternal height (OR 0.95), increased years of formal education (OR 0.94) and no smoking and no drinking (OR 0.73). Variables not significantly associated with a decreased BWZS were the combination of smoking, drinking and history of a previous stillbirth, the combination of drug use with thoughts of self-harm, maternal age, anaemia, gravidity, married or partnered and living together or apart, and possession of a cell phone (Table 2A).

The prevalence rate of newborns with a BWZS < −1.0 in women who did not drink or smoke during pregnancy was 24.5% (Table 2B). Using this as a reference group, the prevalence rate of newborns with a BWZS < −1.0 was not significantly lower in the drinking only group (23.0%; p=0.481), but significantly higher in the smoking only group (29.8%; p=0.016) and in the drinking and smoking group (33.4%; p<0.001) (Table 2B).

Multiple logistic regression of continuous and nominal variables is shown in Table 3. The highest partial ORs were found when there was a history of a previous stillbirth (OR 1.89), cigarette smoking (OR 1.84), hypertension (OR 1.40), education (OR 0.94), BMI (OR 0.95), household income (OR 0.9989), maternal age (OR 1.05), and crowding (OR 1.099).

### Main findings

This study shows that smoking, whether combined with drinking and preeclampsia (OR 2.45), or on its own (OR 1.55), or combined with drinking (OR 1.43), or the absence of smoking (and drinking) (OR 0.73) is significantly associated with low BWZS. Smoking groups have the highest prevalence of low BWZS (22.4%) compared to non-smoking groups (7.4%). A previous stillbirth and preeclampsia on their own were also significantly associated with a low BWZS. In the multiple logistic regression fewer variables seem to have played a role, as the partial ORs were: previous stillbirth (OR 1.89), cigarette smoking (OR 1.84), hypertension (OR 1.40), education (OR 0.94), BMI (OR 0.95), household income (OR 0.9989), maternal age (OR 1.05), and crowding (OR 1.099). This is probably due to internal relationships/correlations among the covariates.

### Strengths and Limitations

The strength of the study is that we collected detailed information on smoking and drinking prospectively on several occasions, obtained information on pregnancy outcome in almost 98%, and used early ultrasound scans to determine the gestational age at birth. We also assessed the influence of mental health and compared the risks when common risk factors were combined.

Limitations are the small numbers of certain variables in different risk factor combinations and using a *z*-score < −1.0, which includes centiles from 0.0 to 15.8, instead of the traditional 10^th^ centile (BWZS −1.285).^22^ However, the risk factors are used as a screening tool only. Our study did not record the specific event associated with self-harm which makes it difficult to confirm direct association.

### Interpretation

Women who smoked and drank and developed preeclampsia had the highest odds for a low BWZS. This confirms a previous finding that cigarette smoking in preeclamptic pregnancies is an additional risk for SGA births.^22^

**Figure 2:**
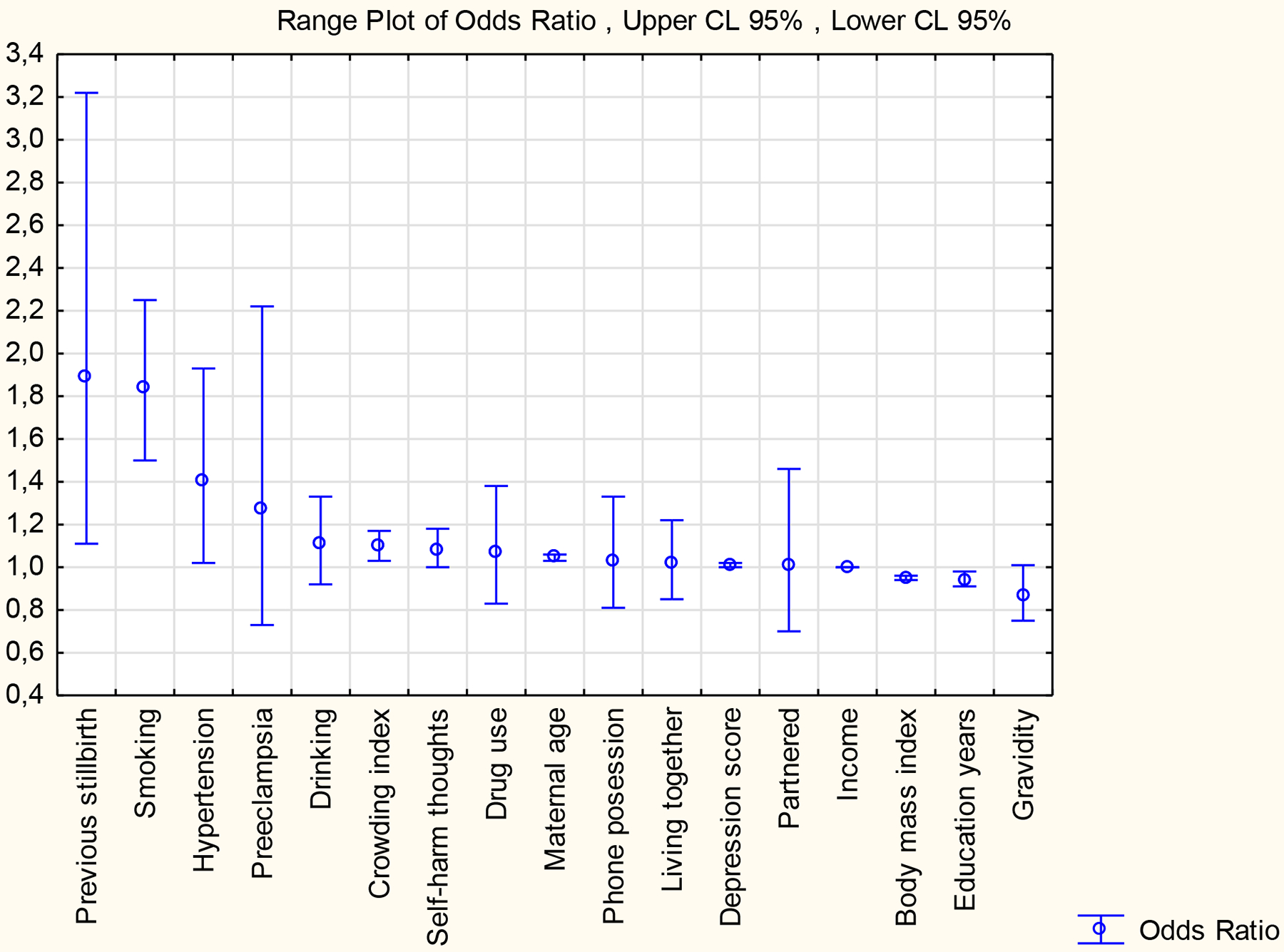
Partial odds ratios of maternal variables compared to low infant birthweight z-scores < −1.0 in multiple logistic regression of continuous and nominal variables

As placental insufficiency is a major cause of stillbirth and contributes to SGA, it seems logical to compare the risks for SGA and stillbirth. In our study, history of a previous stillbirth was the strongest single risk factor for a low BWZS (OR 1.85) (Table 2A), also after logistic regression (OR 1.89) (Table 3, Figure 2). In a multi-country cross-sectional study, Li et al. examined independent and cumulative effects associated with stillbirths in 50 LMICs.^23^ They reported the OR for history of a previous stillbirth (1.55) as their third-highest risk factor, following short maternal height and interpregnancy interval. Several other studies reported increased odds of a stillbirth following a previous stillbirth with ORs ranging between 2.25 and 10.39.^24–27^

Our study has confirmed the well-accepted finding that cigarette smoking during pregnancy is associated with SGA births.^28–31^ The smoking group (OR 1.55) consisted of smokers/non-drinkers and smokers/drinkers while the smoking and drinking group only (OR 1.43) consisted of users of both substances. This may give the impression of a protecting effect of alcohol. However, when we look at the specific groups (Table 2B), the prevalence rate of low BWZS in the smoking only group was 29.8% in contrast to 33.4% when women used both substances (p<0.001). The prevalence of low BWZSs in both smoking groups was 22.4%, compared to 7.4% in both non-smoking groups.

The apparent synergistic effect of alcohol and smoking on fetal growth is difficult to explain. Their combined effects in increasing impedance in the umbilical and uterine arteries could have played a role, but this effect was only seen in heavy drinkers and smokers.^32^ Drinking and smoking are also independent risk factors for oesophageal cancer.^33^ There appears to be a synergistic effect on acetaldehyde (first metabolite of alcohol and a constituent of tobacco smoke) concentrations in saliva.^34^ Synergistic effects of smoking and drinking on stillbirth, preterm birth, and low birthweight (LBW) have previously been reported.^3,35–37^ In addition, it was found that the relative risk of SGA newborns was higher in women who were drinkers and smokers when compared to drinkers who were not smokers,^38^ and that weekday drinkers of 12 g/day or more showed an increased risk in smokers.^39^ Preeclampsia and hypertension are well-known risks for SGA newborns,^40–45^ confirmed in our study (ORs 1.52 and 1.28 respectively).

We found that the use of drugs (9% used marijuana and 4% used methamphetamine as previously reported)^18^ was associated with the fifth-highest individual risk (OR 1.24) for low BWZSs. Other researchers, like Gunn et al.,^46^ confirmed the association of SGA or LBW with marijuana^47–49^ and methamphetamine.^50–52^

We found that prenatal use of alcohol increased the odds for a low BWZS by 18% (Table 2). However, our group of alcohol users consisted of drinkers/non-smokers and drinkers/smokers. For alcohol on its own, no significant difference was found between drinking only (23.0%) and no-drinking-no-smoking (24.5%) with regard to prevalence of low BWZS (p=0.481).

Our finding of an association between alcohol use (non-smokers and smokers in the group) and low BWZS (p=0.01) is supported by other studies which demonstrated a dose-response relationship between alcohol consumption and SGA.^53–56^ In addition, smokers seem to drink more than non-smokers who drink, and drinkers drink more when they are also smokers.^14^ However, a recent systematic review and meta-analysis found no association between alcohol consumption and LBW.^57^

Self-harm ideation alone has inconsistently been independently associated, while it is more often associated with concurrent maternal depressive symptoms that may impact on infant outcome.^58^ Concerningly, our study found that reported thoughts of self-harm during pregnancy staggeringly increased the OR of having an SGA newborn by 13% which is 11% higher than the OR of the EPDS score (Table 2). Although not commonly reported, this finding mimics those of Gandhi et al.^59^ and Czeizel et al.,^60^ who found increased rates of LBW infants among women who had self-harmed during pregnancy. This is speculated to be a result of placental insufficiency induced by the traumatic self-harming event. Our study findings, on the association of self-harming (OR 1.13) and depression in pregnancy (OR 1.02) with a higher occurrence of LBW and SGA, echo the findings of a study in England - where infants born to mothers with a history of prenatal mental illness and behavioural conditions (including self-harm) were >100 g lighter than infants born to women without the maternal vulnerabilities.^61^ Accortt and Schetter^63^ reported significant negative effects of depressive symptoms and endorsement of self-harm on LBW and SGA.^62^ Other studies confirmed the association of depression, with severe depression or depression during mid-pregnancy having the most significant effects.^63,64^ Unsurprisingly, our study reports similar findings to another LMIC setting (India), where the odds of women giving birth to an SGA infant were twice as high for women with a high EPDS score.^64^

The significant partial effect of increased crowding (OR 1.10) and lower education (OR 0.94) is in agreement with many other studies which found that increased crowding,^65–67^ and lower maternal education are associated with increased risks of low BWZS.^68–71^ Our study further confirms that an increased BMI is associated with lower risks of low BWZS newborns (a 5% effect) (Table 3).

## Conclusion

Risk assessment at the beginning of pregnancy should at least include questions on previous stillbirths, use of tobacco, alcohol and drugs during pregnancy, and information on depression, income and education, and measurements of weight and height. Hypertension and preeclampsia should be regarded as additional risks. As up to 20% of pregnancies in LMICs are at high risk,^7,9,10^ careful identification of risks for low BWZS or SGA births and appropriate further management should improve perinatal outcome.

## Figures and Tables

**Table 1:** Basic statistics of cohort

Variable	N	Mean	SD	Median	Minimum	Maximum
Maternal age (years)	5 209	24.4	6.0	23.0	15.0	45.0
Gravidity	5 195	2.1	1.3	2.0	1.0	10.0
Education years	5 202	10.0	1.7	10.0	2.0	13.0
Household income (ZAR)	3 641	862	604.0	750.0	45.5	6 000
Body mass index (kg/m^2^)	5 060	25.1	5.5	23.8	13.7	52.3
Crowding index	4 978	1.6	0.9	1.3	0.3	16.0
Gestational age at birth (days)	5 209	271.8	16.1	275	168	300
Birthweight (gram)	5 209	2905	521	2 970	360	4 000

SD= standard deviation

**Table 2:** Odds ratios of maternal variables compared to low infant birthweight z-scores <−1.0

A. Findings of maternal risk of a birthweight z-score <−1.0		
*Maternal variables*	*Odds ratio (95% Cl)*	*P-value*
Smoking and drinking and preeclampsia	2.45 (1.62–3.72)	0.001
Previous stillbirth	1.85 (1.13–3.00)	0.020
Smoking *	1.55 (1.35–1.77)	0.001
Preeclampsia	1.52 (1.13–2.03)	0.001
Smoking and drinking †	1.43 (1.27–1.62)	0.001
Hypertension	1.28 (1.07–1.53)	0.001
Drug use	1.24 (1.05–1.46)	0.010
Drinking ‡	1.18 (1.04–1.35)	0.010
Thoughts of self-harm	1.13 (1.06–1.21)	0.001
↑ Crowding index	1.10 (1.03–1.17)	0.001
↑ Edinburgh Post natal Depression Scale	1.02 (1.01–1.03)	0.001
Smoking and drinking and previous stillbirth	1.46 (0.73–2.91)	0.29
Drugs and thoughts of self-harm	1.19 (0.92–1.55)	0.18
↑ Maternal age	1.01 (1.00–1.02)	0.17
Anaemia	1.00 (0.89–1.13)	0.98
Gravidity	0.96 (0.92–1.01)	0.09
Married or partnered living together	0.92 (0.82–1.04)	0.20
Married or partnered living apart	0.89 (0.71–1.12)	0.34
Possession of a phone	0.84 (0.70–1.02)	0.07
↑ Household income	0.99 (0.99–0.99)	0.001
↑ Head circumference	0.99 (0.98–0.99)	0.001
↑ Upper arm circumference	0.99 (0.99–1.00)	0.001
↑ Weight	0.98 (0.97–0.98)	0.001
↑ Skinfold thickness	0.98 (0.97–0.98)	0.001
↑ Body mass index	0.95 (0.94–0.97)	0.001
↑ Height	0.95 (0.94–0.96)	0.001
↑ Education years	0.94 (0.91–0.98)	0.001
No smoking and no drinking	0.73 (0.61–0.88)	0.001
B. Birthweight z-score <−1.0 in specific smoking and drinking groups					
*Group (n)*	*Number (%)*	*P-value* ^
No smoking and no drinking group (750)	184 (24.5) ^§^	reference
Drinking only group (851)	196 (23.0) ^¶^	0.481
Smoking only group (888)	265 (29.8) ^¶^	0.016
Drinking and smoking group (2670)	891 (33.4) ^¶^	<0.001

**Table 3:** Partial odds ratios of maternal variables compared to low infant birthweight z-scores <22121.0 in multiple logistic regression of continuous and nominal variables

Significant Variables	Odds Ratio	Lower CL 95%	Upper CL 95%	P-value
Previous stillbirth	1.89	1.11	3.22	0.02
Cigarette smoking	1.84	1.50	2.25	<0.01
Hypertension	1.40	1.02	1.93	0.04
↑ Crowding index	1.10	1.03	1.17	0.004
↑ Maternal age (years)	1.05	1.03	1.06	<0.01
↑ Household income	0.9998	0.9996	0.9999	0.0002
↑ Body mass index	0.95	0.94	0.96	<0.01
↑ Education	0.94	0.91	0.98	<0.01
Non-significant variables	Odds Ratio	Lower CL 95%	Upper CL 95%	P-value
Preeclampsia	1.27	0.73	2.22	0.39
Drinking	1.11	0.92	1.33	0.28
Thoughts of self-harm	1.08	1.00	1.18	0.06
Drug use during pregnancy	1.07	0.83	1.38	0.62
Possession of a phone	1.03	0.81	1.33	0.79
Living together	1.02	0.85	1.22	0.84
↑ Edinburgh Postnatal Depression Scale	1.01	1.00	1.02	0.15
Significant Variables	Odds Ratio	Lower CL 95%	Upper CL 95%	P-value
Married or partnered	1.01	0.70	1.46	0.95
↑ Gravidity	0.87	0.75	1.01	0.07
